# Ferroptosis-Related Long Noncoding RNAs as Prognostic Biomarkers for Ovarian Cancer

**DOI:** 10.3389/fonc.2022.888699

**Published:** 2022-06-09

**Authors:** Kaili Wang, Shanshan Mei, Mengcheng Cai, Dongxia Zhai, Danying Zhang, Jin Yu, Zhexin Ni, Chaoqin Yu

**Affiliations:** ^1^ Department of Traditional Chinese Gynecology, The First Affiliated Hospital of Naval Medical University, Shanghai, China; ^2^ Department of Gynecology of Traditional Chinese Medicine, Shanghai University of Traditional Chinese Medicine, Shanghai, China; ^3^ International Peace Maternity and Child Health Hospital, School of Medicine, Shanghai Jiao Tong University, Shanghai, China

**Keywords:** ferroptosis, long noncoding RNA, biomarkers, tumor immune, ovarian cancer

## Abstract

Ovarian cancer (OC) is a highly malignant gynecologic tumor with few treatments available and poor prognosis with the currently available diagnostic markers and interventions. More effective methods for diagnosis and treatment are urgently needed. Although the current evidence implicates ferroptosis in the development and therapeutic responses of various types of tumors, it is unclear to what extent ferroptosis affects OC. To explore the potential of ferroptosis-related genes as biomarkers and molecular targets for OC diagnosis and intervention, this study collected several datasets from The Cancer Genome Atlas-OC (TCGA-OC), analyzed and identified the coexpression profiles of 60 ferroptosis-related genes and two subtypes of OC with respect to ferroptosis and further examined and analyzed the differentially expressed genes between the two subtypes. The results indicated that the expression levels of ferroptosis genes were significantly correlated with prognosis in patients with OC. Single-factor Cox and LASSO analysis identified eight lncRNAs from the screened ferroptosis-related genes, including lncRNAs RP11-443B7.3, RP5-1028K7.2, TRAM2-AS1, AC073283.4, RP11-486G15.2, RP11-95H3.1, RP11-958F21.1, and AC006129.1. A risk scoring model was constructed from the ferroptosis-related lncRNAs and showed good performance in the evaluation of OC patient prognosis. The high- and low-risk groups based on tumor scores presented obvious differences in clinical characteristics, tumor mutation burden, and tumor immune cell infiltration, indicating that the risk score has a good ability to predict the benefit of immunotherapy and may provide data to support the implementation of precise immunotherapy for OC. Although *in vivo* tests and research are needed in the future, our bioinformatics analysis powerfully supported the effectiveness of the risk signature of ferroptosis-related lncRNAs for prognosis prediction in OC. The findings suggest that these eight identified lncRNAs have great potential for development as diagnostic markers and intervention targets for OC and that patients with high ferroptosis-related lncRNA expression will receive greater benefits from conventional chemotherapy or treatment with ferroptosis inducers.

## Introduction

Ovarian cancer (OC) is the seventh most common cancer of the female reproductive system and one of the most common gynecologic malignant tumors worldwide, and the 5-year survival rate of OC has remained between 30% and 50% for decades (1, 2). The female ovary is located deep in the pelvic cavity and has multiple histological characteristics, causing a series of clinical diagnostic problems for OC. Effective diagnostic methods for early OC remain lacking. According to its histopathological characteristics, OCs are categorized as epithelial OC tumors, germ cell tumors, or sex cord-stromal tumors, accounting for more than 90%, 2–3%, and 5–6% of cases, respectively (3). The preventive measures and treatments for OC, such as surgical treatment, systemic treatment, and targeted therapy, have made great progress (4). However, due to the lack of typical clinical symptoms in the early stage of onset, 75% of cases are diagnosed at stage III or IV, and 70–80% of patients relapse after early treatment (5). Most OC cases identified late in life initially respond well to chemotherapy treatment, but the development of resistance always leads to a poor long-term prognosis (6). Therefore, it is urgent to identify sensitive biomarkers to provide personalized diagnosis and accurate prognostic evaluation for the treatment of patients with OC (7, 8), as has occurred with the emergence of platinum resistance and new anticancer therapies, such as immunotherapy (7, 8).

Ferroptosis is a newly identified nonapoptotic, iron-dependent form of programmed cell death that is characterized by the accumulation of lipid peroxidation; it was originally found by Dixon et al. in 2012 (9). As accumulating reactive oxygen species (ROS) attack the polyunsaturated fatty acids (PUFAs) of phospholipid membranes with multiple unsaturated double bonds, they trigger nonenzymatic lipid peroxidation and produce end-products of lipid peroxidation, such as 4-hydroxy-2-nonenol and malondialdehyde (10), which have toxic effects on cells, thereby initiating iron ion-dependent cell death (11), that is, ferroptosis. Emerging evidence suggests that ferroptosis may be a critical adaptive process for eradicating carcinogenic cells (12; Wang et al., 2021). It may also play vital roles in the pathological development and clinical therapeutics of tumor cells and cancers relevant to ferroptosis, p53, noncoding RNAs (ncRNAs), and the tumor microenvironment (TME); moreover, a series of small molecules have been found to be able to induce ferroptosis in a wide range of cancer cells. However, whether ferroptosis and its related genes and proteins are involved in the formation and growth of OC is still unclear (13–15).

LncRNAs, which are RNA molecules with more than 200 nucleotides, have been demonstrated to have a potential role in regulating normal or cancerous cells: on the one hand, their dysregulation results in abnormalities in cell migration, proliferation, the cell cycle, apoptosis and autophagy, which are closely involved in different cancers; on the other hand, alterations in lncRNAs lead to the emergence of cancer (16, 17; Li and Ugalde et al., 2021; 18, 19). For instance, the lncRNAs NEAT1, SNHG3 and H19 can function in cancer proliferation and metastasis as upstream mediators of the STAT3 pathway (16); lncRNAs HANR, BORG, etc., drive DOX resistance by activating the NF-κB, PI3K/Akt, and Wnt pathways; and the lncRNA SOX2OT-V7 can activate protective autophagy in response to the stress caused by DOX (17, 18). In OC, it has been found that the lncRNAs ROR, HOTAIR, H19 and UCA1 can influence the progression of ovarian cancer by promoting EMT (20), while the upregulation of the lncRNA CCAT2 significantly accelerates the proliferation, migration and invasion of tumor cells in OC (21). In addition, it has been found that lncRNA SPRY4-IT1 can promote OC by affecting the cell cycle. Therefore, regulating the expression of lncRNAs is of significant importance in cancer therapy.

Multiple studies have shown that lncRNAs may regulate cancers through ferroptosis. For instance, the lncRNA NEAT1 can regulate ferroptosis sensitivity (22), LINC00336, as a competing endogenous RNA, can inhibit ferroptosis in lung cancer (Wang Z et al., 2021), and LINC00618 can reduce the expression of lymphoid-specific helicase, thus inhibiting ferroptosis (Wang et al., 2021). Mao et al. demonstrated that the cytoplasmic lncRNA P53RRA is downregulated and interacts with Ras-GTPase activating protein binding protein 1 (G3BP1) to transfer p53 from the G3BP1 complex, resulting in p53 retention in the nucleus, leading to ferroptosis (23). Furthermore, GABBB1 and its antisense lncRNA GAPB1-AS1 can interact in erastin-induced ferroptosis (24).

In addition, recent studies have shown that lncRNAs directly or indirectly regulate ferroptosis and its related signaling pathways (25) and play a key role in the processes of ferroptosis-regulated cancers (23, 24; Wang et al., 2020; 26). On the one hand, the expression of lncRNA TINCR increases in breast cancer, and lncRNA FTX promotes the proliferation and invasion of gastric cancer through miR-144/ZFX (27, 28). TINCR combines with STAU1 to guide STAU1 to regulate the stability of OAS1, and low levels of OAS1 aggravate tumor proliferation and migration (29). The lncRNA LINC00336 may increase the growth of lung cancer cells through the LSH/ELAVL1/LINC00336 axis, accelerate tumor formation, and inhibit ferroptosis pathways in lung cancer cells (30, 31).

On the other hand, lncRNAs are widely involved in p53-related signaling pathways. p53 promotes dipeptidylpeptidase-4 (DPP4) translocation into the tumor cell nucleus in a transcription-independent manner, forming a p53-DPP4 complex, and thus negatively regulates the ferroptosis of colorectal cancer cells by inhibiting the association of DPP4 and nicotinamide adenine dinucleotide phosphate oxidase 1 (NOX1) (32). Furthermore, lncRNAs inhibit the binding of miRNAs and mRNAs by competitively adsorbing miRNAs in response to the original, resulting in the silencing of target genes (33). The above studies have shown that lncRNAs may be key molecular regulators in tumor and ferroptosis pathways and participate in the occurrence and development of tumors. A recent study found that eight lncRNAs, including AC138904.1, AP005205.2 and UBXN10-AS1, which are associated with iron metabolism in OC, were strongly associated with the overall survival of patients (34). However, lncRNAs involved in regulating ferroptosis pathways have not been reported in the diagnosis, intervention or prognosis of OC; it is still unclear whether these ferroptosis-related lncRNAs play roles in OC.

**Figure 1:**
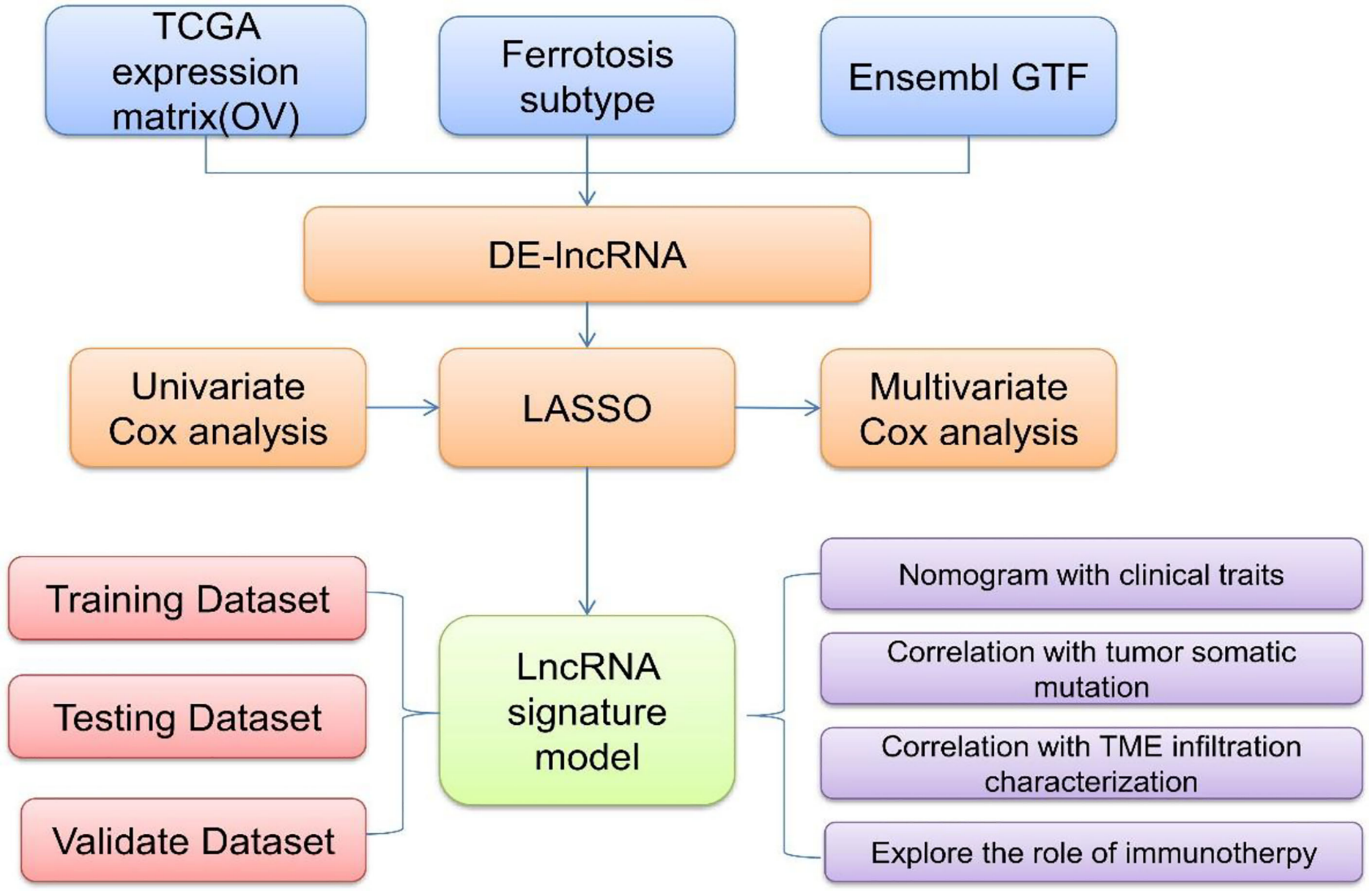
A flowchart and analysis approaches of this study.

To explore and verify whether ferroptosis-related lncRNAs are involved in the pathogenesis and disease processes of OC and further validate their significance in the diagnosis and prognosis evaluation of OC, the expression profile and clinical follow-up information data of patients with OC were obtained and analyzed in the TCGA database (**Figure 1**). Differentially expressed ferroptosis-related lncRNAs were screened, and the lncRNAs that were significantly coexpressed with ferroptosis-related genes were finally identified by single-factor Cox and least absolute shrinkage and selection operator (LASSO). Using the TCGA-OC data, a risk scoring model between the identified lncRNAs and OC was also constructed with multivariate Cox regression to assess and confirm the accuracy of patient diagnosis and prognosis prediction. TCGA-OC tumor samples were divided into two different groups on the basis of the risk score, and differences in clinical characteristics, tumor mutation burden, and tumor immune cell infiltration (ICI) were found. Moreover, the ability of the risk score to predict the benefit of immunotherapies for OC was further evaluated. These results (**Figure 1**) provide powerful support for the implementation of precise immunotherapy for OC and act as a reference for the early diagnosis and prognosis evaluation of patients with OC. Furthermore, these ferroptosis-related lncRNAs and genes may be vital regulatory molecules for OC treatment.

## Methods

### Samples and Data Collection for Clinical Patients With OC

The gene expression profile data and clinical follow-up information of patients with OC (**Figure 1**) were collected by exploring and analyzing the TCGA database (https://portal.gdc.cancer.gov/). Then, the obtained samples and RNA-Seq data were analyzed and processed as follows: (1) samples without clinical follow-up information were removed; (2) samples with a certain survival time, less than 30 days of survival, or no survival status were removed; (3) the peaks of gene expression assays were converted to Gene Symbols; (4) probes that corresponded to multiple genes were removed; and (5) the expression of genes corresponding to multiple Gene Symbols was presented as the average value. In this study, data for a total of 357 tumor samples were acquired and confirmed from TCGA-OC. The clinical statistics of the samples are shown in **Table 1**.

**Table 1:** Profile data of gene expression and clinical follow-up information from TCGA-OC.

		TCGA-OC
Survial		
OS	Status_0	139
	Status_1	218
Age		
	Age>65	112
	Age<=65	245
Grade		
	G1	1
	G2	45
	G3	310
	G4	1
Stage		
	Stage_I	1
	Stage_II	21
	Stage_III	283
	Stage_IV	52

### Heterogeneous Clustering Analysis of Tumorous Gene Profiles Related to Ferroptosis

As ferroptosis is activated by excessive lipid peroxidation, accompanied by the accumulation of lipid peroxidation markers, the cell death caused by ferroptosis could be completely suppressed by the consumption of iron chelators, lipophilic antioxidants, lipid peroxidation inhibitors, and polyunsaturated fatty acids. Ferroptosis is reflected by metabolic dysfunction involving abnormal levels of ROS, iron, and PUFAs around tumors. Thus, in this study, various genes and pathways related to iron, energy metabolism, lipid synthesis, and oxidative stress were considered, as they may negatively affect the sensitivity of ferroptosis-related analyses.

Research papers on ferroptosis published in the past 3 years were collected, and then all of the ferroptosis-related genes in these research results were obtained. Using an impact factor (IF) above 10 as an inclusion criterion to select high-quality studies, a total of 60 ferroptosis-related genes were identified (10, 35–37). The expression profiles of these 60 screened genes were extracted from the TCGA database. Unsupervised clustering was performed on Consensus Cluster Plus-R packages *via* Euclid with the Ward’s and PAM methods. The procedure was repeated 1000 times to ensure test stability.

### Differentially Expressed Genes Between the Two Subtypes

On the basis of the differentially expressed genes and clustering analysis results (**Figure 1**), tumorous samples from patients with OC were divided into two subtype groups (Fer-1 and Fer-2). Then, the differentially expressed genes between the two groups was compared and analyzed *via* the limma package of R software. After the filtering threshold of the differentially expressed gene was adjusted to P < 0.05 and | log2 (fold change) | > 2/3, the genome annotation file in Ensemble was used to extract the lncRNAs from the set of differentially expressed genes.

### Feature Dimension and Risk Scoring Model for Ferroptosis-Related lncRNAs

On the basis of the two subtypes of lncRNAs, tumor risk scoring models were constructed for the screened ferroptosis-related LncRNAs (**Figure 1**). First, the single-factor Cox algorithm was used to reduce the size of the lncRNA gene sets related to ICI subtypes to reduce noise and eliminate redundant genes. Afterward, LASSO (Tibshirani) was used to screen variables to reduce the number of genes in the risk models. Finally, a multifactor Cox regression model was used to construct a risk scoring model for tumor ICI. The calculation formula was as follows:


Risk_scores=∑Coef(i)*Exp(i)

### Gene Set Enrichment Analysis (GSEA)

GSEA is a gene enrichment analysis method that takes a knowledge-based approach to interpreting genome-wide expression profiles. One or more functional gene sets, stemming from the gene matrix transposition file formats, were sorted according to the correlation levels between the gene expression values and tumorous phenotypes, thus forming the different gene lists (**Figure 1**). They were then used to judge whether the genes of each gene set were enriched in the upper or lower part of the gene list to assess the influence of the coordinated phenotypic changes.

### Statistical Analysis

Statistical analysis was performed in R software (3.6). R packages and tools used in the study were indicated. Statistical methods were described in the corresponding sections. P < 0.05 was considered as significant. ns, no significance. *P < 0.05, **P < 0.01, ***P < 0.001, ****P < 0.0001.

## Results

### Classification of OC Based on Ferroptosis-Related Gene Sets

**Figure 2:**
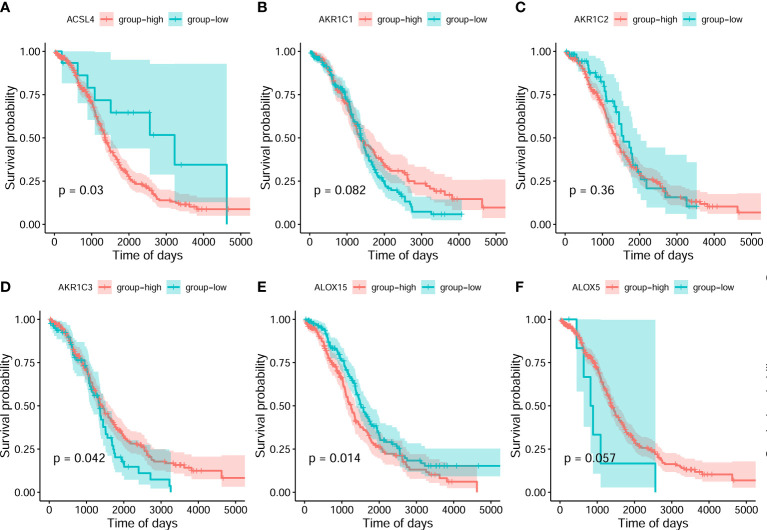
Classification analysis for 6 ferroptosis-related gene expression and overall survival curve in the OC gene dataset of the Cancer Genome Atlas. **(A)** ACSL4 **(B)** AKR1C1 **(C)** AKR1C2 **(D)** AKR1C3 **(E)** ALOX15 **(F)** ALOX5.

On the basis of the best density algorithm, 60 ferroptosis-related genes for OC were divided into high- and low-expression groups. The high-expression group included *AKR1C3, CD44, CISD1, GCLC, SLC7A11, PHKG2, HSBP1, KEAP1, NQO1, SLC1A5, G6PD*, and *PGD.* The low-expression group included *ACSL4, ALOX15, ALOX12, ATP5MC3, CBS, FANCD2, CRYAB, LPCAT3, STEAP3, ACACA, ZEB1, NOX1, ABCC1, IREB2, HMOX1*, and *ACSF2*. Both groups showed significant differences between the prognostic treatment survival curves of OC (p<0.05, **Figure 2** and **Supplementary Material F1**).

**Figure 3:**
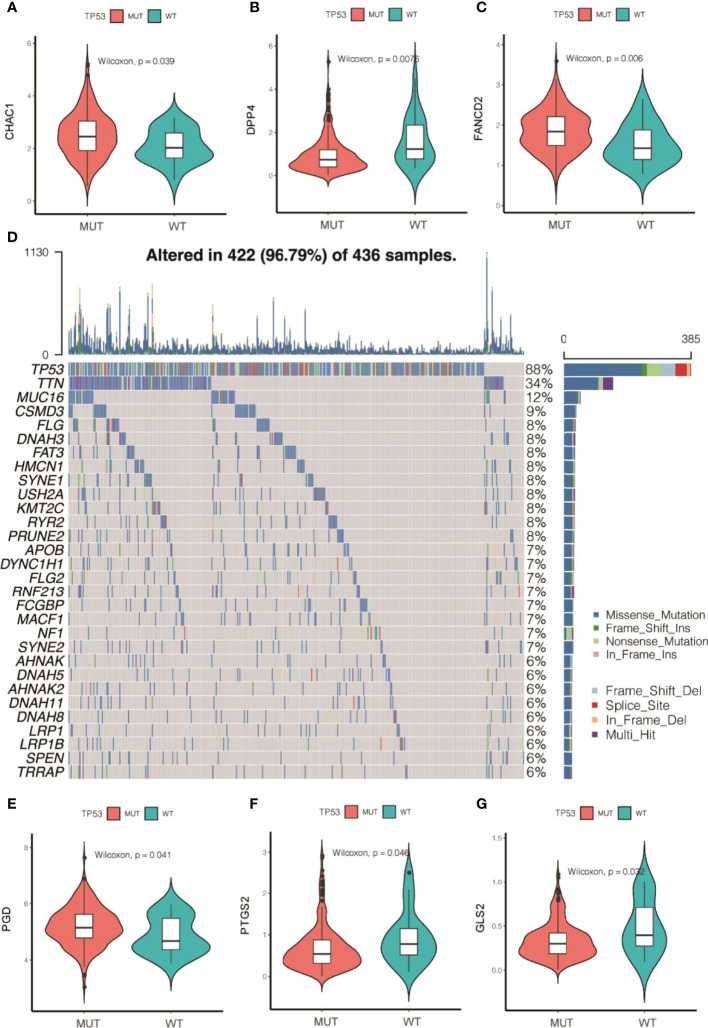
Gene mutations of tumor samples in OC gene dataset of the Cancer Genome Atlas and its regulation on 60 ferroptosis-related genes. **(A)** Waterfall chart of genetic mutations; **(A–G)** effects of TP53 mutations on the expression of ferroptosis-related genes.

**Figure 4:**
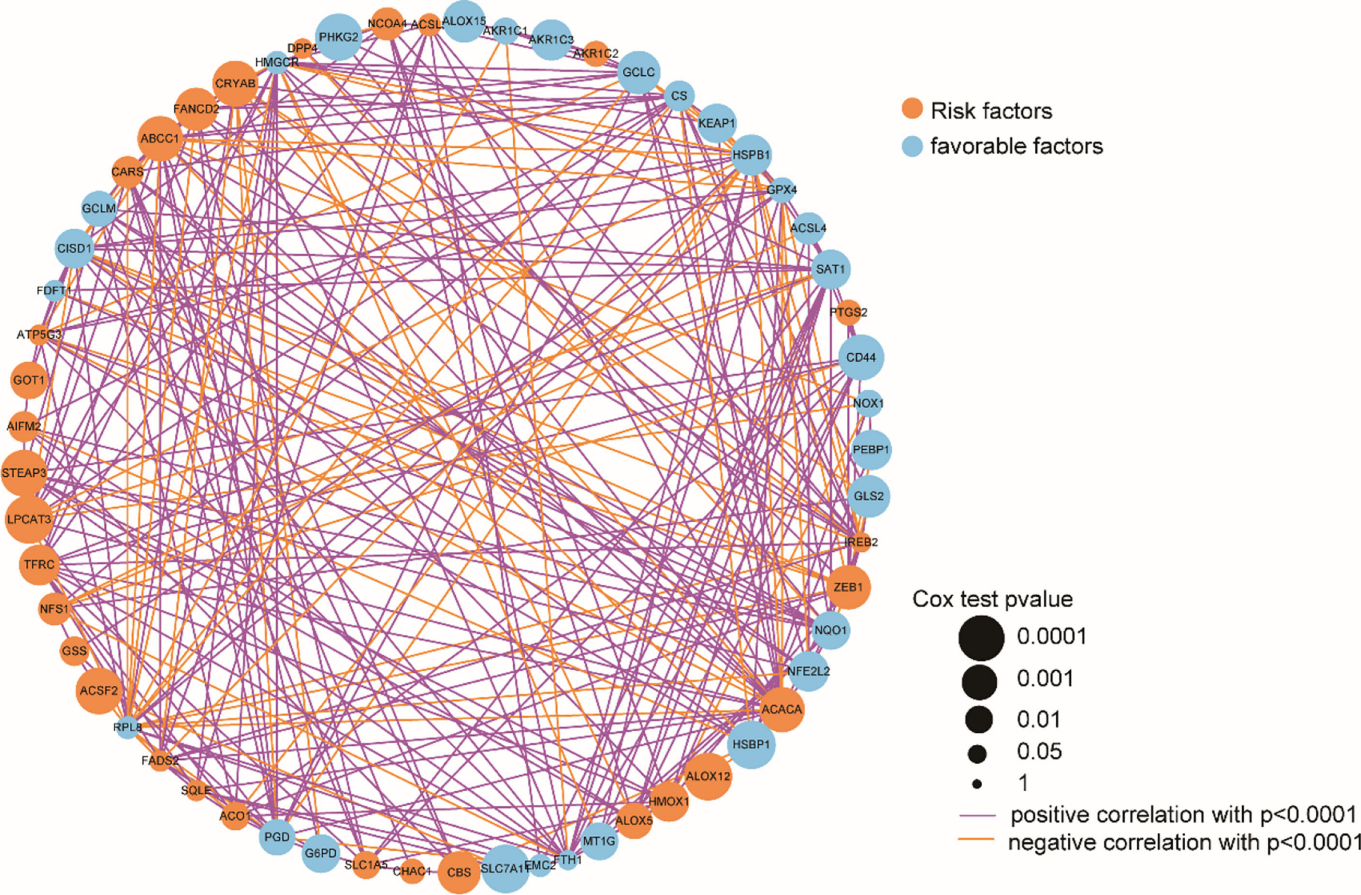
Correlation of 60 ferroptosis-related gene expression.

Subsequently, the analyzed results suggested that 96.79% of tumor samples in the TCGA-OC dataset had gene mutations. Among them, TP53 and TTN mutations were most prevalent, occurring in up to 88% and 34% of the samples, respectively (**Figure 3A**). Hypothesis tests were performed to further determine whether the gene mutations in TP53 and TTN affected the expression of 60 ferroptosis-related genes. The results showed that in the TP53-mutation group, the expression levels of CHAC1, FANCD2, PGD, and other genes were significantly higher than in the wild type, whereas the expression levels of ALOX5, DPP4, GLS2, and FTH1 were significantly lower (**Supplementary Material F2**). In the TTN-mutation group, the expression of CHAC1 and KEAP1 was significantly lower than in the wild type, while the expression of NCO4A and ACO1 was significantly higher (**Figure 3** and **Supplementary Material F2**). Furthermore, the expression levels showed mutual promotion effects and a high correlation of expression among the 60 ferroptosis-related genes (**Figure 4**)

### Functional Annotations of Significant Genes for Ferroptosis Subtypes

**Figure 5:**
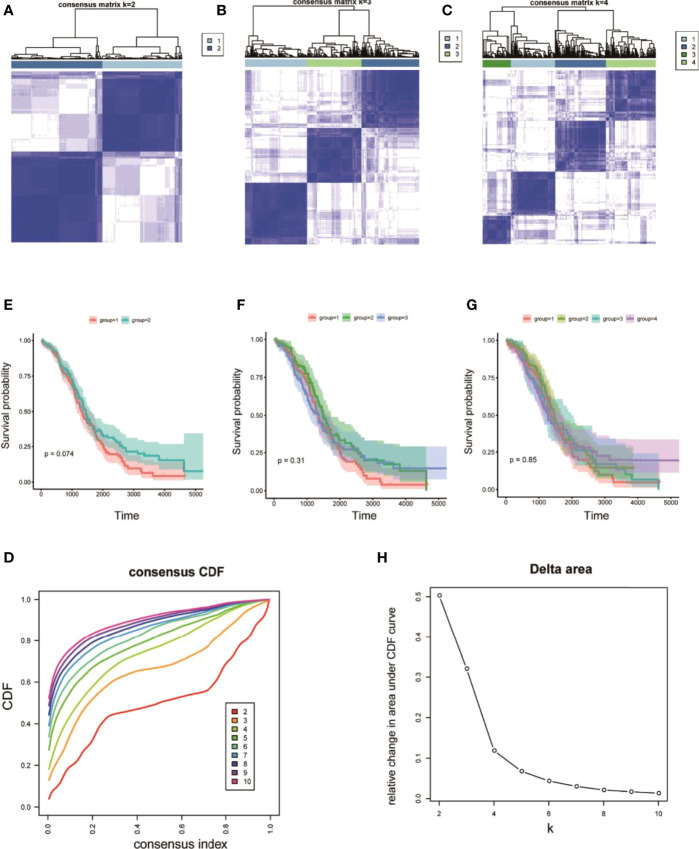
Heterogeneous clustering analysis for 60 ferroptosis-related gene expression values. **(A–C)** Clustering results, with classification k values of 2, 3, and 4, respectively; **(D)** CDF curve distribution of consistent clustering; **(E–G)** survival analysis of OC samples, with classification k values of 2, 3, and 4, respectively; **(H)** distribution of AUC under the CDF curve of consistent clustering.

On the basis of the expression values of the 60 screened ferroptosis-related genes, clustering analysis suggested that when the cluster k values were 2, 3, or 4 (**Figures 5A–C**), but especially 2, the difference between the two subtypes was significant, and the trend was proximate (**Figures 5A–D**). Meanwhile, analyses of survival rates indicated that when k = 2, survival was distinct between the two subtypes. Fer-2 predicted better prognosis than Fer-1, and Fer-1 was associated with poor prognosis. These results confirmed the two subtypes of OC with respect to ferroptosis, Fer-1 and Fer-2.

**Figure 6:**
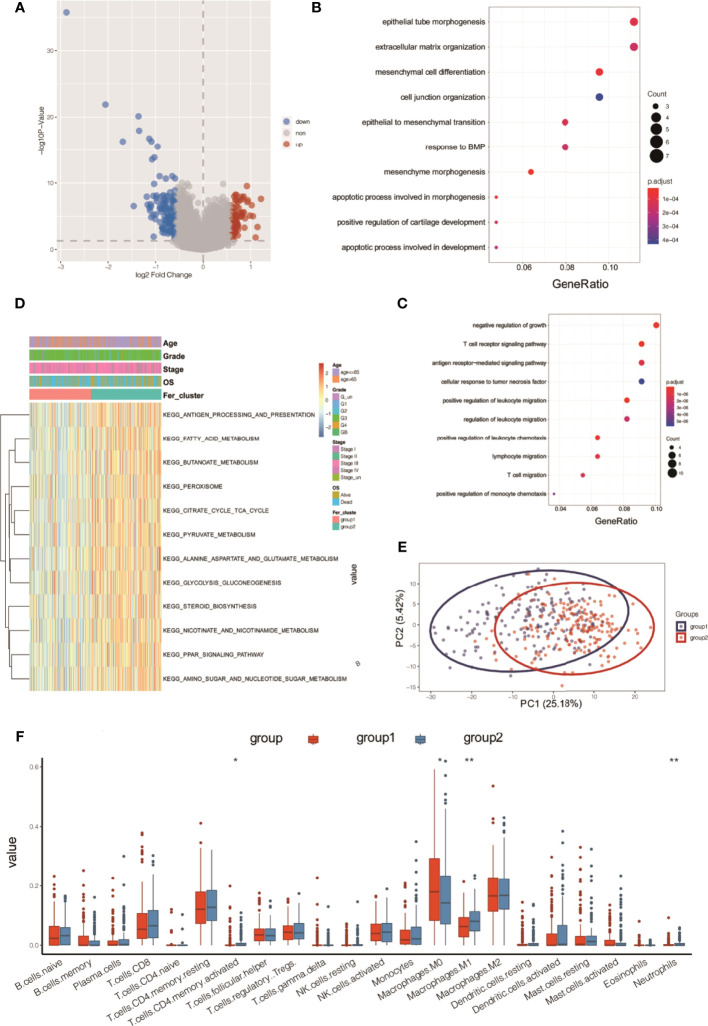
Functional annotation analysis of differentially expressed genes beween two ferroptosis subtypes in OC tumors. **(A)** Volcano map of differentially expressed genes between two subtypes; **(B)** bubble chart of high-expression genes in Fer-1; **(C)** bubble chart of high-expression genes in Fer-2; **(D)** KEGG analysis; **(E)** PCA analysis; **(F)** tumor ICI values of the two groups. *P < 0.05, **P < 0.01.

The R limma package was used to analyze the differences in the gene expression levels in the two subtypes of Fer-1 and Fer-2. When the gene expression threshold values were set to adjusted values of p < 0.05 and | log2 (fold change) | > 0.58, 207 differentially expressed genes were identified (**Table S1**). Of these, 78 genes had higher expression levels in Fer-1 than in Fer-2 (**Figure 6**), whereas 129 genes had lower expression levels. Functional enrichment analysis of GO annotations was used for the genes that were differentially expressed in Fer-1 and Fer-2 (**Figure 6B**). The Fer-1 genes may be principally engaged in the enrichment biological processes of mesenchyme morphogenesis, mesenchymal cell differentiation, and apoptotic process involved in morphogenesis. The Fer-2 genes may be related to the positive regulation of leukocyte migration, the T-cell receptor signaling pathway, and the positive regulation of leukocyte chemotaxis (**Figure 6C**).

In addition, enrichment analysis suggested that these differentially expressed genes were involved in the KEGG pathways shown in **Figure 6D**, including fatty acid metabolism, butanoate metabolism, pyruvate metabolism, glycolysis, gluconeogenesis, steroid biosynthesis, nicotinate and nicotinamide metabolism, PPAR signaling, and amino sugar and nucleotide sugar metabolism.

Principal component analysis (PCA) of the differentially expressed gene profiles indicated that the first principal component accounted for 25.18% of the difference between subtypes, and the Fer-1 and Fer-2 subtype samples were clearly separated (**Figure 6E**). The immune infiltrating cell amounts and ratios showed significant differences between the Fer-1 and Fer-2 groups, such as in central memory CD4+-T cells, activated CD4+-T cells, M0 macrophages, and M1 macrophages, indicating significant differences in the immune microenvironment between the two ferroptosis subtypes related to OC (**Figure 6F**). In summary, consistency could be observed between the gene expression and prognosis profile of the two ferroptosis subtypes in OC tumors, indicating that the classification method was sound and reasonable.

### Risk Score of Ferroptosis-Related lncRNAs in OC

Pearson correlation analysis was used to identify the lncRNAs that were coexpressed with ferroptosis-related genes (P < 0.001 and |R| >0.4) in OC to explore the expression of ferroptosis-related lncRNAs and their role in overall survival in OC. The results showed that 215 lncRNAs had significant coexpression with ferroptosis genes (**Table S2**).

**Figure 7:**
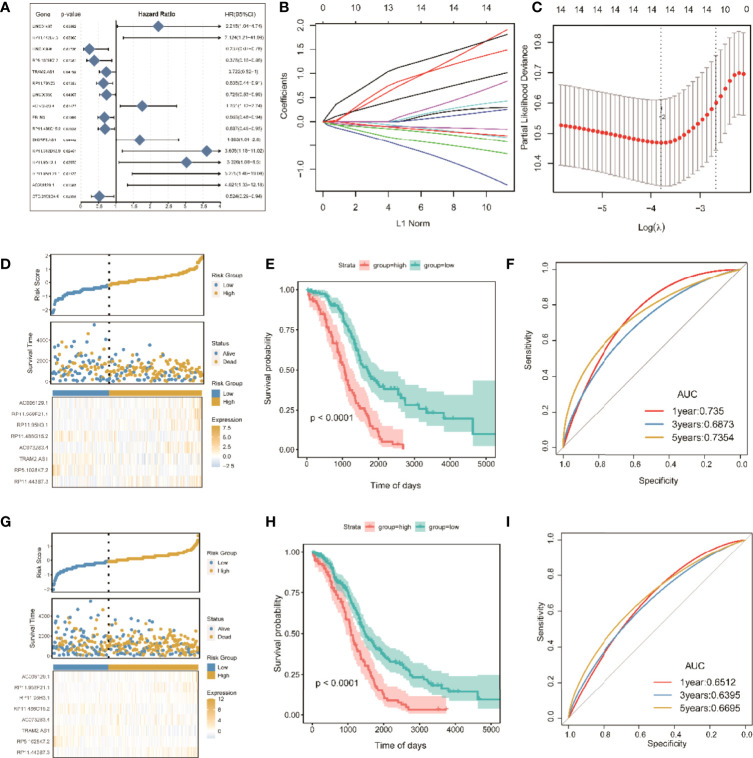
Screening of significant LncRNAs in OC and construction of its risk score model. **(A)** Forest plots of single-factor Cox analysis; **(B)** change trajectory of each independent variable, the horizontal axis represents the log value of the independent variable lambda (the vertical axis represents the coefficient of the independent variable); **(C)** confidence interval under each lambda; **(D)** risk distribution map of the risk score in the training set; **(E)** survival curves of risk score in the training set; **(F)** ROC curves of risk score in the training set; **(G)** distribution map of risk score in all sets. **(H)** risk score survival curve in the total set; (I) ROC curve of risk score in total set.

A risk scoring model of tumor ICI was further established on the basis of the coexpressed lncRNAs. First, the overall TCGA-OC set (n = 368) was divided into a training set (n = 246) and a test set (n = 122). In the training set, single-factor Cox analysis was used to identify 215 candidate lncRNAs. When the significance threshold was set to P < 0.05, 16 lncRNAs were retained and confirmed (**Figure 7A** and **Table S3**).

To improve clinical application, LASSO Cox regression, as a compressed estimation method, was used to screen variables *via* the R package for glmnet (**Figures 7B, C**). With the gradual increase in lambda, the number of independent variable coefficients trending to zero gradually increased. Through 10-fold cross-validation, the optimal lambda of 0.06048449 was chosen for the model. Thus, 14 lncRNAs were retained as the target genes for the next step (**Table S4**).

Finally, the Akaike information criterion (AIC) was used for further stepwise regression to obtain a sufficient degree of fitting with fewer parameters. The regression model finally reduced 14 lncRNA genes to eight, namely, lncRNAs RP11-443B7.3, RP5-1028K7.2, TRAM2-AS1, AC073283.4, RP11-486G15.2, RP11-95H3.1, RP11-958F21.1, and AC006129.1 (**Table S5**). Multivariate Cox regression was also used to construct a risk scoring model of the eight lncRNAs related to tumor ICI in OC as follows: risk score = (2.65865) * RP11-443B7.3 + (−1.82366) * RP5-1028K7.2 + (−0.43758) * TRAM2-AS1 + (1.01663) * AC073283.4 + (−0.49744) * RP11-486G15.2 + (1.34710) * RP11-95H3.1 + (1.97023) * RP11-958F21.1 + (1.44802) * AC006129.1.

The samples were divided into high-risk and low-risk groups on the basis of the algorithm of optimal density gradient to further assess the effects of the established risk score on overall survival (OS, **Figure 7D**). The results showed that the proportion of nonsurviving patients was higher in the high-risk group than in the low-risk group. The Kaplan–Meier analysis also showed that the OS of the high-risk group was significantly shorter than that of the low-risk group (**Figure 7E**), suggesting that the risk score possesses great potential for the prognosis of OS in the TCGA-OC sets. The AUC values of prognostic sensitivity at 1, 3, and 5 years were 0.735, 0.6873, and 0.7354, respectively. Similarly, the OS time of patients in the high-risk group was significantly lower (**Figure 7G**), and the AUC values at 1, 3, and 5 years were 0.6512, 0.6395, and 0.6695, respectively.

### Relationships of Risk Score and Clinical Pathological Features for Patients

**Figure 8:**
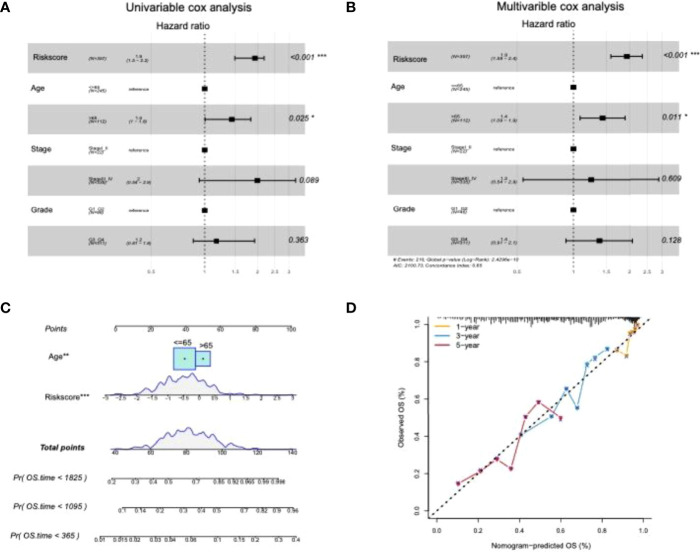
Prognostic predictive assession of risk score and clinical pathological features for patients with OC. **(A, B)** Univariate and multivariate cox analysis of clinical variables and clinical pathological features; **(C)** nomogram between clinical features and risk score; **(D)** calibration chart of the nomogram at 1, 3, and 5 years. P < 0.05 was considered as significant. *P < 0.05, ***P < 0.001.

The age and tumor grades of clinical characteristics are important clinical diagnostic and treatment indicators for patients with OC. It is beneficial for OC diagnosis and intervention to assess and verify the relationships between the prognostic risk scores of the identified lncRNAs and the clinical characteristics. Univariate and multivariate Cox analyses of clinical variables showed that the risk score was an independent prognostic factor among patients stratified by age, OC stage, and grade (**Figures 8A, B**). Subsequently, multivariate Cox analysis was used to construct a nomogram between risk score and age for convenient use in the clinic (**Figure 8C**). Furthermore, the calibration curves of the nomograms showed that the risk score had prognostic stability for OC (**Figure 8D**).

**Figure 9:**
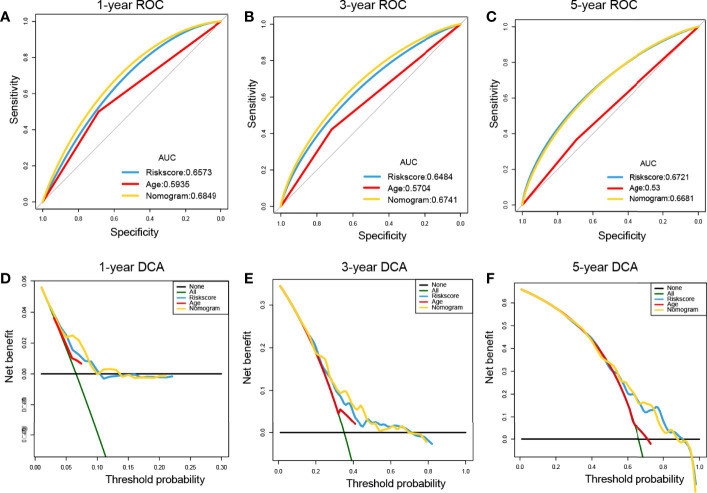
Prognostic performance assessment of risk score. **(A–C)** ROC curves of risk score nomograms at 1, 3, and 5 years, respectively; **(D–F)** DCA distribution maps at 1, 3, and 5 years, respectively.

ROC curves showed that the nomogram of the risk score had higher prognostic predictive performance (above 0.65) at 1, 3, and 5 years than other indicators (**Figures 9A–C**). DCA was carried out to further determine the accuracy of the nomograms. The net benefits of the nomogram at 1, 3, and 5 years were obviously and significantly higher than those of the other clinical variables (**Figures 9D–F**). All of these results confirmed that the tumor risk score was a relatively independent prognostic indicator for OC.

### Relationships of Risk Score and Tumor Mutational Burden (TMB) for OC

**Figure 10:**
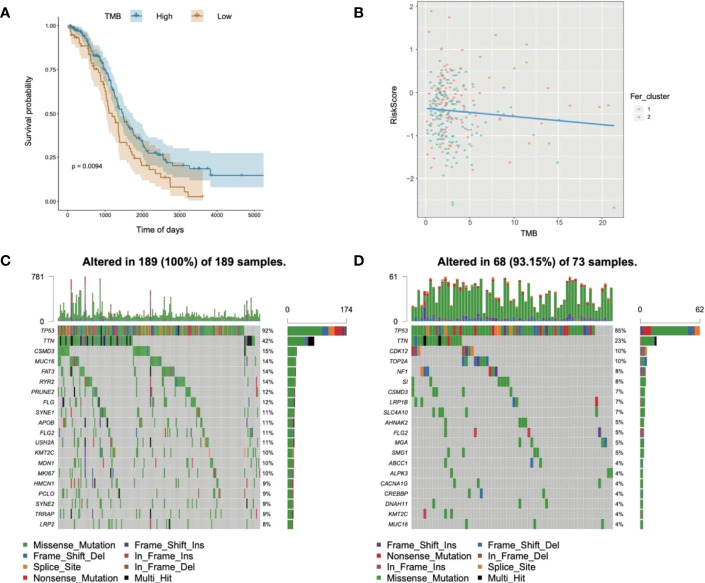
Relationships of risk score and tumor mutational burden for OC. **(A)** Survival curves of high- and low-TMB groups; **(B)** correlation analysis in two groups; **(C)** waterfall chart of gene mutation in the high-risk score group; **(D)** waterfall chart of gene mutation in the low-risk score group.

Substantial clinical evidence suggests that TMB may partly determine individual responses to cancer immunotherapy. Thus, exploring the intrinsic links between TMB and risk score and elucidating the genetic characteristics of each ferroptosis subgroup are of great importance. The R package Survminer was used to further calculate and test the optimal density gradient threshold of TMB and survival. The obtained tumor samples were divided into high- and low-TMB score groups, and a significant difference in survival was found between the two groups (**Figure 10A**). Subsequently, correlation analysis showed that the risk score was negatively correlated with TMB (R = −0.09), as shown in **Figure 9B**. Patients with high TMB had a lower risk score, suggesting better treatment effects of immunotherapy on patients with OC with high TMB scores (**Figure 10B**).

In addition, to evaluate the somatic variant distributions associated with immunity, the top 20 genes with high-frequency variants in the OC-actuated genes were compared between the low- and high-risk score groups (**Figures 10C, D**). Analysis of mutation annotation files from the TCGA-OC sets revealed significant differences in the mutational spectrum between the low- and high-risk score groups. These results may provide new guidance for exploring the mechanisms of ferroptosis gene mutation-related immune checkpoints.

### Correlation of Tumor Risk Score and ICI

**Figure 11:**
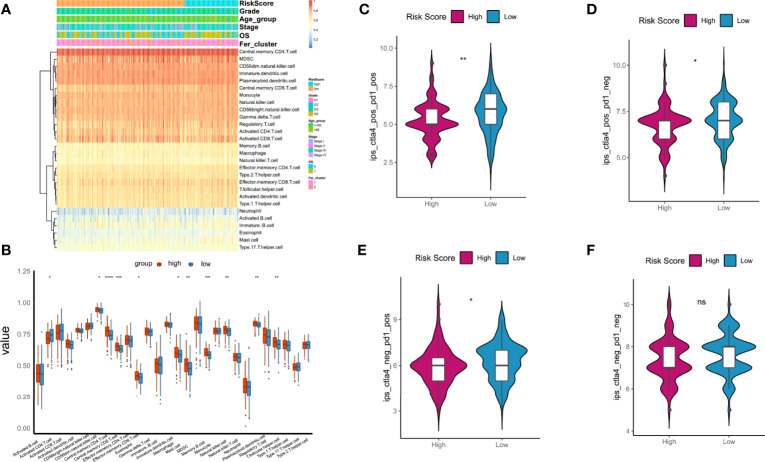
Relationships between tumor risk score and immune cell infiltration (ICI). **(A)** Heatmap of distribution of ICI ratios; **(B)** boxplot of differences in ICI between high- and low-risk score groups; **(C–F)** relationships between tumor risk score and related immunophenoscore. P < 0.05 was considered as significant. ns, no significance. *P < 0.05, **P < 0.01, ***P < 0.001, ****P < 0.0001.

GSEA was used to evaluate 28 kinds of ICIs in the TCGA-OC datasets to explore the relationships between the risk score constructed by tumorous ferroptosis-related lncRNAs and tumor immune microenvironments (**Table S6**). Regarding the overall levels of tumor ICI, immune cells with high-level infiltration included CD4^+^-central memory T cells, CD56^+^-suspicious natural killer cells (CD56^+^-NK cells), immature dendritic cells, myeloid suppressor cells (MDSCs), and CD8^+^-central memory T cells (**Figure 11A**). Significance analysis showed that in the high-risk score group, the levels of infiltrating central memory CD4^+^-T cells, CD8^+^-T cells, and MDSCs significantly increased compared with those in the low-risk group. In contrast, the level of infiltrating activated CD4^+^-T cells was significantly lower than that in the low-risk group (**Figure 11B**).

The immunotherapy cohort was evaluated and analyzed on the basis of the immunophenoscore (IPS) in TCGA-OC samples to further confirm the prediction performances and efficiency of the tumor risk score on the immunotherapy benefits of patients with OC. As shown in **Figures 10C–F**, four types of IPS, ips_ctla4_neg_pd1_neg, ips_ctla4_pos_pd1_neg, ips_ctla4_neg_pd1_pos, and ips_ctla4_pos_pd1_pos, were significantly higher in the low-risk score group than in the high-risk score group, indicating that patients in the low-risk score group were more likely to benefit from immunotherapies.

## Discussion

OC develops to advanced stages without producing symptoms, and it is characterized by extensive metastasis, chemoresistance, and poor prognosis. Currently, platinum and paclitaxel drugs are the main clinical chemotherapies for OC, but limited or little progress can be observed in the prognosis for advanced OC. Recent evidence has shown that because ferroptosis can be induced in cancer cells, the enhancement of cancer cell sensitivity to ferroptosis is obviously beneficial for cancer treatment (38–40). In this study, we obtained OC-related expression profile data and clinical follow-up information. A total of 60 ferroptosis-related genes were identified (**Figures 2**, **4**), and a heterogeneous cluster analysis was performed on the basis of the expression values of the 60 identified genes (**Figure 5**). Preclinical- and clinical-related research results indicate that ferroptosis is involved in the proliferation, migration, infection, and apoptosis of cancer cells (32, 41). Thus, the present study supported the speculation that ferroptosis-related genes or lncRNAs may play a vital role in the treatment and prognosis of OC. Moreover, the two molecular subtypes were found to have significant differences: the prognosis of Fer-2 was better than that of Fer-1, and Fer-1 showed poor prognosis (**Figure 5**).

Previous studies found that the overexpression of lncRNA RP11 inhibited the proliferation, migration, colony formation, and nuclear translocation of SOX2 in OC cells. The lncRNA RP11 also exerted inhibitory effects on tumor growth in nude mice and could exert tumor suppressor effects by regulating the RP11-PAK2-SOX2 axis in OC (42). Consistent with these previous results, our study screened and identified eight ferroptosis genes as potential biomarkers of OC diagnosis, treatment, or prognosis, namely, lncRNA RP11-443B7.3, RP5-1028K7.2, TRAM2-AS1, AC073283.4, RP11-486G15.2, RP11-95H3.1, RP11-958F21.1, and AC006129.1 (**Figure 6** and **Tables S3**– and **S5**). This finding suggested that the present research has great clinical predictive value and application prospects and that lncRNAs may be used as potential treatment and prognostic biomarkers for OC.

*In vitro* cell experiments showed that the expression of lncRNA RP11 was negatively correlated with treatment time and the dose of cisplatin administered (43). Western blot analysis results indicated that cisplatin induced autophagy in OC cells in a time- and dose-dependent manner, and cisplatin combined with lncRNA RP11 markedly decreased the autophagy of OC cells, increased apoptosis, and inhibited their cellular activities. In addition, cisplatin could induce autophagy in OC cells (44, 45). Overexpression of the lncRNA RP11 improved the autophagy induced by cisplatin, thereby enhancing the effect of cisplatin on OC cells (43).

This was confirmed by our work, in which a tumor risk scoring model was constructed for ICI on the basis of the coexpressed lncRNAs with ferroptosis-related genes in OC (**Figure 7**). Thus, the lncRNA RP11 and the other lncRNAs coexpressed with ferroptosis-related genes identified through model analysis in the present study may be vital genes and potential regulatory targets for OC.

Clinical research data further confirmed the above results. Serum samples were collected from clinical patients with OC, those with benign ovarian disease, and controls. Then, the differential gene expression levels in the serum were detected using RT–PCR methods (46). The results showed that serum lncRNA RP5 expression was significantly upregulated in the malignant tumor group compared with the benign and control groups. The lncRNA RP5 expression was lower in malignant ovarian tissue and adjacent nontumor tissues from patients with OC than in normal tissues. Furthermore, the operator characteristic curves showed that the expression values of lncRNA RP5 obviously distinguished the patients with OC from patients with benign masses and control patients, with an accuracy rate of 96% (46). These results indicate that these new diagnostic biomarker RNAs could help diagnose OC and could be of great importance for the early detection and treatment of clinical tumors. The eight identified lncRNAs related to ferroptosis are likely to be key biomarkers for the diagnosis and prognosis of OC.

YAP is a transcriptional cofactor that binds to thousands of enhancer sites to stimulate tumor aggressiveness (47). Based on whole-genome chip analysis, TRAM2 was identified as the YAP-bound enhancer, and the target genes of the lncRNA TRAM2 had the strongest correlation with YAP-expression activities in almost all tumors. Interestingly, the lncRNA TRAM2 affected the cell proliferation, migration, and invasion phenotypes induced by YAP, and it was associated with low survival rates in patients with tumors. Therefore, the lncRNA TRAM2 may be a crucial mediator of YAP-induced tumorigenicity (Li et al., 2021).

Recent studies indicated that the lncRNA AC, as a competitive endogenous RNA (ceRNA), played a key role as a tumor suppressor gene in breast cancer tissues and exerted its anti-breast cancer effect by regulating the expression of miR-18b-5p and targeting the cell division factor DOCK4 (48–51). In breast cancer cells, the lncRNA AC was used as a ceRNA to regulate miR-18b-5p and inhibit its negative regulatory effects on the expression of targeted DOCK4 genes (48–51). In addition, lncRNA AC decreased the migration ability of breast cancer cells, inhibited cell cycle progression from G0/G1 phase to S phase, enhanced the apoptosis of breast cancer cells, and reversed the EMT phenotype (52). Therefore, abnormal changes in lncRNAs are closely related to the phenotypes of tumor cell proliferation, migration, and invasion, and they are important molecular targets for tumor regulation. In the pathological process of the induction and occurrence of OC, it is urgent to determine whether lncRNAs play a key role, and their relationship with ferroptosis remains unclear (53, 54). In contrast, a risk scoring model of the screened lncRNAs related to tumor ICI was used to solve the current problems (**Figure 7**). Kaplan–Meier analysis was implemented to analyze the difference in risk score between the two groups, and the OS of the high-risk group was found to be significantly lower than that of the low-risk group (**Figures 7E–G**). These results indicated that the risk score had high performance in predicting OS in the TCGA-OC dataset.

Moreover, univariate and multivariate Cox analyses were performed on the risk score and important clinical variables (**Figure 7**). The tumor samples in TCGA-OC were divided into high- and low-risk score groups on the basis of the risk score. The differences in clinical characteristics (**Figures 8**, **9**), tumor mutation burden (**Figure 10**), and tumor ICI (**Figure 11**) between the two risk groups in OC were deeply explored and illustrated, and the predictive ability of the risk score with respect to the benefits of immunotherapy were further evaluated, thus providing powerful support for the implementation of precise immunotherapy for OC.

In summary, multiple bioinformatics methods were used in this study to screen and identify ferroptosis-related genes and eight important lncRNAs in OC, namely, lncRNAs RP11-443B7.3, RP5-1028K7.2, TRAM2-AS1, AC073283.4, RP11-486G15.2, RP11-95H3.1, RP11-958F21.1, and AC006129.1 (**Table S5**). The modeling results confirmed that these lncRNAs were closely related to OC. These ferroptosis-related genes and important lncRNAs may serve as vital clinical biomarkers, and targeting them may be a potential approach for the diagnosis, clinical treatment, and prognostic evaluation of OC (55–57). Our work suggests that patients with high ferroptosis-related lncRNA expression will be benefit more from conventional chemotherapy or treatment with ferroptosis inducers. However, this study has some shortcomings and limitations. First, due to the limited number of OC samples in the database, more database information and patient data need to be included in future research to validate the current findings. Second, the eight screened ferroptosis genes were identified and confirmed only through bioinformatics analysis in the later stages. *In vivo* and *in vitro* OC models should be designed to further verify and clarify the clinical prognosis associated with these ferroptosis-related genes and molecules and their potential intervention effects in OC.

## Data Availability Statement

The original contributions presented in the study are included in the article/**Supplementary Material**. Further inquiries can be directed to the corresponding authors.

## Author Contributions

Conceptualization: ZN and CY; Visualization: KW, SM, and MC; Writing-original draft preparation: KW, JY, SM, and MC; Writing-review and editing: JY, ZN, DXZ, DYZ, and CY; Supervision: JY, ZN, and CY. All authors agree to be accountable for the content of the work. All authors contributed to the article and approved the submitted version.

## Funding

This work was funded by National Natural Science Foundation of China (82074206, 81973896), Shanghai 3-Year Action Plan for Traditional Chinese Medicine [ZY(2018-2020)-FWTX-1107], Leading Project of Traditional Chinese Medicine of Shanghai Science and Technology Committee (19401930200), and China Postdoctoral Science Foundation (2020M681337).

## Conflict of Interest

The authors declare that the research was conducted in the absence of any commercial or financial relationships that could be construed as a potential conflict of interest.

## Publisher’s Note

All claims expressed in this article are solely those of the authors and do not necessarily represent those of their affiliated organizations, or those of the publisher, the editors and the reviewers. Any product that may be evaluated in this article, or claim that may be made by its manufacturer, is not guaranteed or endorsed by the publisher.
