# Supplementary material for: Ferroptosis-Related Long Noncoding RNAs as Prognostic Biomarkers for Ovarian Cancer
Source: Front Oncol. 2022 Jun 9;12:888699. doi: 10.3389/fonc.2022.888699 (PMC9218568; doi:10.3389/fonc.2022.888699)
Supplement: Supplementary file 1 [file DataSheet_1.pdf]

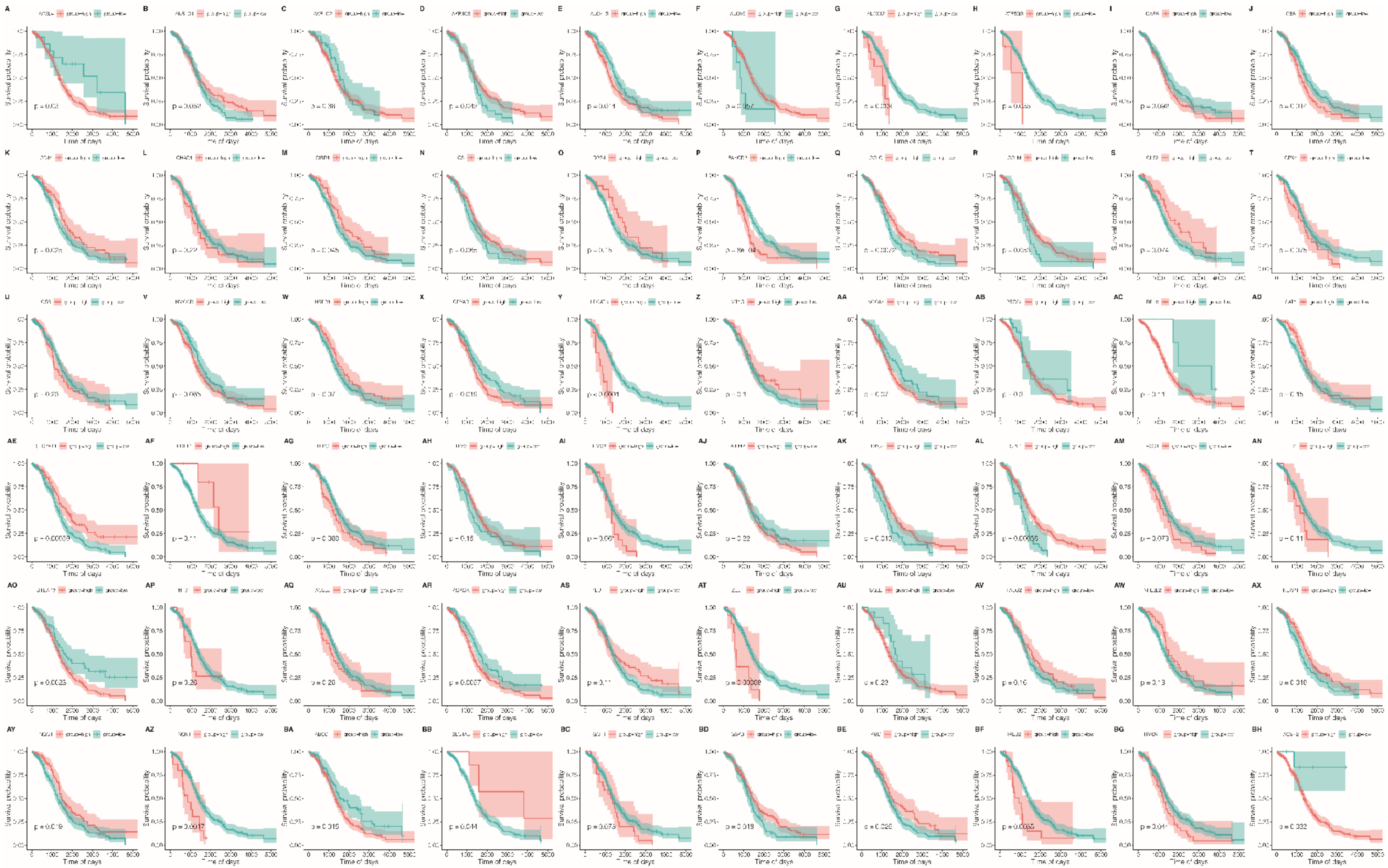

Supplementary materials F1 the extended results of 60 ferroptosis-related gene expression and the overall survival curve in the TCGA-OC data.

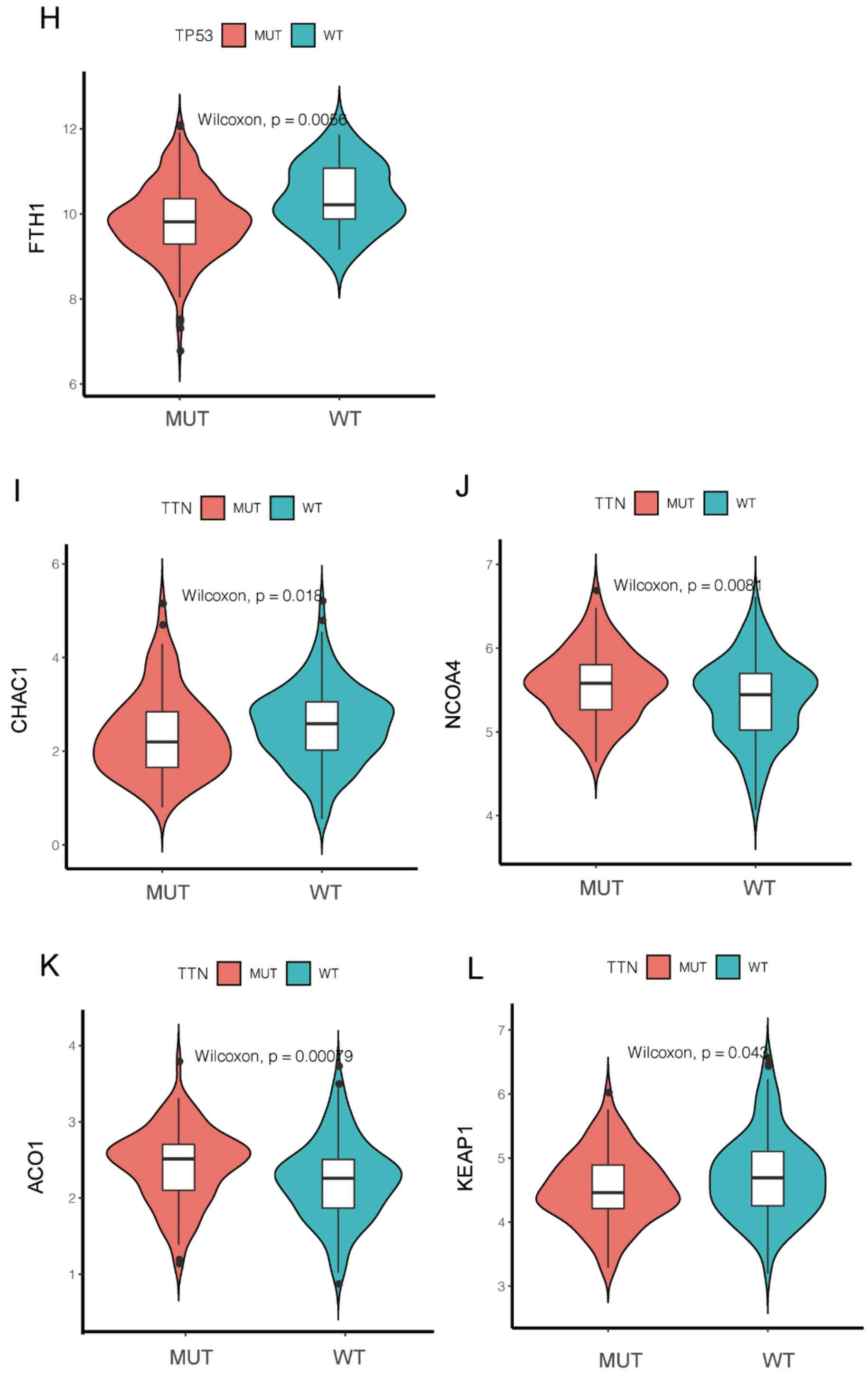

Supplementary materials F2 Gene mutations of tumor samples in the TCGA-OC data and its regulation on 60 ferroptosis-related genes. (A) Waterfall chart of genetic mutations; (A-H) effects of TP53 mutations on the expression of ferroptosis-related genes; (I-L) effects of TP53 mutations on the expression of ferroptosis-related genes.

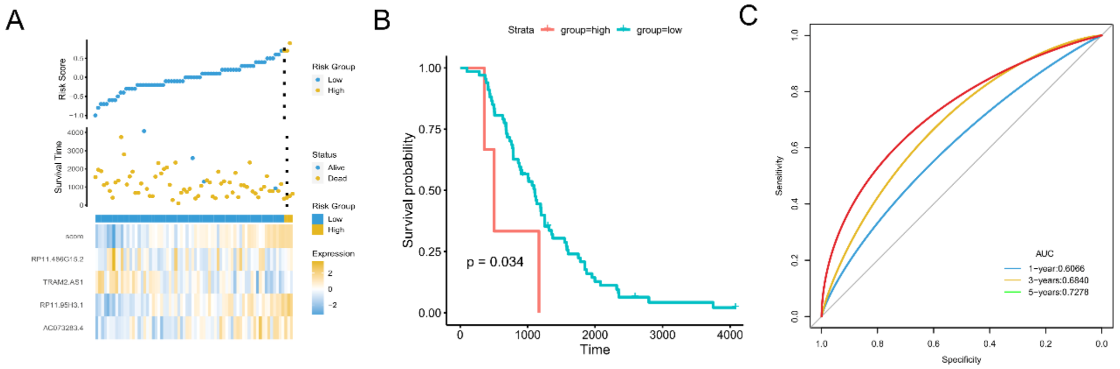

Supplementary materials F3. Further validation verification of the this in another OC tumor dataset.
